# Agenda

**DOI:** 10.1080/13814788.2017.1310518

**Published:** 2017-06-29

## 2017

**Table:** 

June	
**28-01 (July), Prague, Czech Republic**. ***Wonca Europe*** http://www.woncaeurope2017.eu	**Theme: General Practice in Europe: growing together in diversity** ‘United in Diversity’ is the official motto of the European Union. Having been already united in WONCA family we want to grow as a discipline in European diversity by learning from each other and exchanging experience and knowledge. This explains a theme of our Conference: ‘Growing together in Diversity.’ It allows us to open all dimensions of the discipline.

**Table:** 

October	
**19-22, Dublin, Ireland**. ***EGPRN Autumn meeting****(European General Practice Research Network* http://www.egprn.org	**Theme: Environmental Health**

## 2018

**Table:** 

May	
**24-27, Krakow, Poland.** ***Wonca Europe*** http://www.woncaeurope2018.eu	**Theme:** Quality, Efficiency, Equity

**Table:** 

June	
**30 (May)-02, Porto, Portugal.** ***9th IPCRG World conference*** http://www.theipcrg.org	**Theme: Respiratory Health: Adding Value in a Resource Constrained World**

## 2019

**Table:** 

June	
**26-29, Bratislava, Slovak Republic** ***Wonca Europe*** http://www.woncaeurope2019.org	**Theme: General Practice: The human side of Medicine**

